# Registered Nurse Staffing and Inpatient Outcomes in Korean Long-Term Care Hospitals

**DOI:** 10.3390/healthcare12242509

**Published:** 2024-12-11

**Authors:** Sujin Shin, Jung Min Yoon, Eun-Ju Moon, Mi-Ji Lee, Jin-Hwa Park

**Affiliations:** 1 College of Nursing, Ewha Womans University, Seoul 03760, Republic of Korea; ssj1119@ewha.ac.kr (S.S.); jmyoon@ewha.ac.kr (J.M.Y.); miji_lee@naver.com (M.-J.L.); 2 College of Nursing, Daegu Catholic University, Daegu 42472, Republic of Korea; quffndi@naver.com

**Keywords:** nurse staffing, long term care, hospitals, outcomes, inpatients

## Abstract

Background/Objectives: There is a relative lack of specific research on registered nurse (RN) staffing in long-term care hospitals in the Republic of Korea. This study investigated the association between RN staffing levels and inpatient outcomes in long-term care hospitals in the Republic of Korea. Methods: Nationwide data of long-term care hospitals from the Health Insurance Review and Assessment Services website were used to analyze the association between registered nurse staffing levels and 7 inpatient outcome indicators. Results: The results indicated that in long-term care hospitals with higher RN staffing levels, there was an improvement in moderate-to-severe pain, activities of daily living enhancement, lower prevalence of indwelling catheters, reduction in the incidence of pressure ulcers, improvement in existing pressure ulcers, and increased rate of return to the community. Similar results were observed in analyses conducted according to disease classification groups. Conclusions: These findings suggest that increasing RN staffing levels can enhance patient safety and improve treatment outcomes. Furthermore, this study provided important foundational data for developing policies to optimize RN staffing in long-term care hospitals in the Republic of Korea.

## 1. Introduction

As of 2022, 9.7% of the global population was aged 65 and older. This percentage is expected to increase to 11.7% by 2030 and 16.4% by 2050 [1]. The older population in the Republic of Korea is increasing at a much faster rate than in other countries. As of 2023, population in the Republic of Korea, aged 65 years and older, comprises approximately 18.4% of the total population. Projections indicate that by 2025 this demographic will represent 20.6% of the population, categorizing the Republic of Korea as a super-aged society. By 2040, 34.4% of the population is expected to belong to this age group [2]. Accompanying the increase in this older population, the prevalence of geriatric and chronic diseases necessitating long-term hospitalization has influenced the demand for medical services provided by long-term care hospitals (LTCHs). At the end of 2022, there were 1435 LTCHs in the Republic of Korea, constituting 34% of all hospital-level medical institutions [3]. This number has significantly increased from 867 in 2010, which almost doubled within a decade. Concerns have been raised regarding the quality of service in nursing hospitals due to this rapid quantitative growth.

In the Republic of Korea, hospitalization costs in LTCHs are reimbursed under a per-diem system based on resource consumption, which is part of the Prospective Payment Systems. Based on the severity of a patient’s condition, daily hospitalization fees are categorized into five levels, with different rates applied accordingly [4]. The per-diem reimbursement system, which is effective in reducing medical costs that tend to increase in fee-for-service models, has drawbacks. This per-diem reimbursement system can incentivize minimization of resource input, which might lead to the underprovision of services [5]. To address these drawbacks, the Korean government has implemented quality assessments of LTCHs since 2008. These assessments apply quality indicators to encourage autonomous quality improvements in LTCHs by grading them based on the total scores. However, because the total score of such an evaluation is simply the sum of each indicator score, there are limitations to assessing inpatient outcomes based on the level of nurse staffing, which comprises approximately 57% of the medical workforce in labor-intensive LTCHs [6].

Nursing-sensitive patient outcomes refer to variables representing patient results influenced by interventions provided by the nursing staff [7,8]. A previous study used variables such as the prevalence of physical restraints, urinary tract infections, indwelling catheter use, monthly incidence of new pressure ulcers, falls, and recreational activities as indicators of patient outcomes in LTCHs [9]. Registered nurses (RNs), who are professionals primarily responsible for assessing changes in patient conditions, can be considered to have the most critical impact on patient health outcomes.

The level of RN staffing is a key factor influencing nursing-sensitive patient outcomes. The staffing levels of RNs in LTCHs have direct effects on improvements in activities of daily living (ADL) [10]. In long-term institutional care, increased contact hours with highly educated nursing staff have been associated with decreases in the prevalence of urinary tract infections and less catheter use [11], and higher RN hours per resident day positively affects weight loss and pain [12,13]. These findings demonstrate that an adequate RN staffing level is a crucial factor in improving various inpatient outcomes, thereby significantly enhancing the quality of care in LTCHs.

In the Republic of Korea, the standards for nurse staffing in LTCHs are distinct from those of other hospital-level institutions. The Medical Law allows up to two-thirds of the nursing quota to be fulfilled by certified nursing assistants instead of RNs. As a result, there is significant variability in the level of RNs across LTCHs. Since 2008, the government has provided financial incentives based on nurse staffing levels in all hospitals with inpatient wards. In 2019, these incentives were enhanced, introducing additional bonus payments for institutions with a skill mix of more than two-thirds of nurses to encourage higher RN staffing levels in LTCHs. Despite these efforts, empirical studies on the effectiveness of these policies remain limited, highlighting the need to examine the association between RN staffing levels and inpatient outcomes in Korean LTCHs following the changes in these policies.

Previous research identified several factors that influence patient outcomes, including the type of establishment, hospital location, and market characteristics. Compared with non-private facilities, private equity-owned nursing homes have higher prevalence rates of pressure ulcers [14] and patient mortality [15]. Significant regional differences in the decline in ADL and the occurrence of pressure ulcers have also been reported between metropolitan and nonmetropolitan areas [16]. Additionally, increased competition among hospitals has been associated with positive outcomes such as lower rates of patient mortality, readmissions, and adverse effects from medications and procedures [17]. These variables must be considered when examining the association between RN staffing levels and inpatient outcomes.

This study analyzed national data in the Republic of Korea to assess the associations between RN staffing levels and inpatient outcomes and evaluate the effects of policies related to RN staffing levels. Furthermore, the associations between RN staffing levels and inpatient outcomes according to LTCH disease classification groups to reflect the inpatient case mix were investigated.

## 2. Materials and Methods

### 2.1. Design

This retrospective study conducted a secondary data analysis to assess the association between RN staffing levels and inpatient outcomes in LTCHs.

### 2.2. Data Source

All the participants of this study were all in LTCHs in the Republic of Korea that claimed per-diem fixed fees, established before 1 July 2021, and had been operating for at least 6 months as of 31 December 2021. In the Health Map provided by Health Insurance Review and Assessment Service (HIRA), information such as hospital details, results of quality assessment data, and medical fees are publicly disclosed to the citizens through the “Find a Good Hospital in Our Area” service. Data for this study were collected from July to August 2023 from LTCHs whose quality assessment results are publicly available on HIRA website. Data analyses were conducted using 1243 LTCHs that received grades based on the quality assessment results from 1265 LTCHs.

This study was exempted from review by the Institutional Review Board of Daegu Catholic University because the data used are publicly available on the HIRA website in the Republic of Korea (CUIRB-2023-E005).

### 2.3. Measures

The variables used for analyses and their definitions are described in Table S1. The RN staffing levels were determined using data on the number of inpatients per RN. For inpatient outcomes, seven quality indicators were used: the proportion of patients with pain improvement, improvement in ADL, urinary catheters, newly developed pressure ulcers, improved pressure ulcers, weight loss of 5% or more compared with the previous month, and the rate of return to the community. Hospital- and market-related factors were covariates and used as control variables.

### 2.4. Data Analyses

The analyses were conducted using SAS version 9.4 (SAS Institute Inc., Cary, NC, USA). Descriptive statistics such as frequency, percentage, mean, standard deviation, and minimum and maximum values were used to describe general characteristics, RN staffing levels, and inpatient outcomes. Additionally, this study analyzed the disease codes of the top three most prevalent diseases among hospitalized patients in each LTCH, along with their respective percentages, as publicly available on the HIRA website.

Based on the Medical Law Enforcement Regulations [18], which specify inpatient target groups for LTCHs (patients with 1. geriatric diseases, 2. chronic diseases, and 3. in the recovery period after surgeries or injuries) and considering prior research [19] that used inpatient case mix classifications, we classified the disease codes into disease groups. These disease classification groups were discussed among the researchers and finalized after consulting with experts; they are listed in Table S2.

The association between RN staffing levels and inpatient outcomes was analyzed after controlling for hospital- and market-related factors. The analysis model used was a generalized estimating equation Poisson regression with a log (bed) offset utilizing PROC GENMOD.

## 3. Results

### 3.1. General Characteristics of LTCHs, RN Staffing Levels, and Inpatient Outcomes

Among the 1243 LTCHs analyzed, 628 (50.5%) were privately owned. The average number of beds per hospital was 195; 699 institutions (56.2%) had more than 100 beds but fewer than 200 beds. In the 1229 LTCHs that reported patient ADL, the average percentage of patients capable of performing daily activities independently (level 1) was 9.64%. Those needing some assistance (level 2) comprised 43.85%, whereas 46.47% of the patients were entirely dependent on others (level 3). Grade 2 was the most common LTCH evaluation grade from the quality assessments of 408 institutions (32.8%). For reference, grade 1 represents the best-performing hospitals, while grade 5 indicates areas needing significant improvement. The most frequently reported diseases per hospital based on Korean Standard Classification of Disease, 8th Revision (KCD-8) were dementia in Alzheimer’s disease (G30), hemiplegia, cerebral infarction, Parkinson’s disease, and emergency use of code U07. The KCD-8 is used for medical coding, research, and statistical analysis in healthcare settings, and it is established based on the framework of the International Classification of Diseases, 10th Revision (ICD-10) published by the World Health Organization (WHO), adapted to fit the healthcare system and conditions of the Republic of Korea [20].

The market-related factor measured by the Herfindahl-Hirschman Index indicated an average competition intensity of 163.80 among LTCHs within a given area. When categorized by location, areas outside capital cities and metropolitan areas comprised the largest group, with 480 LTCHs (38.6%).

The RN staffing level had an average of 9.95 inpatients per RN, ranging from a minimum of 2.60 to a maximum of 29.20 inpatients per RN. During the 2021 survey period, 66.25 ± 26.97% of inpatients in LTCHs experienced improvement in moderate or severe pain compared to the previous month. Additionally, 28.91 ± 25.23% of the inpatients showed improved ability to perform daily activities compared to the previous month. The percentage of inpatients with an indwelling urinary catheter during the survey period was 3.62 ± 6.58%. The percentage of inpatients who developed new pressure ulcers compared to the previous month was 0.10 ± 0.26%, whereas 39.69 ± 22.43% of inpatients demonstrated improvement in pressure ulcers. The percentage of inpatients who experienced more than 5% weight loss compared to the previous month was 0.37 ± 1.13%. A total of 48.92 ± 16.85% of inpatients were discharged back to the community during the survey period (Table 1).

The disease classification groups based on the most frequently reported diseases in LTCHs were neurological diseases, dementia, and infections (Table S2). The disease classification groups representing more than 10% of the total were used for additional analyses.

### 3.2. RN Staffing Level and Inpatient Outcomes for LTCHs

After controlling for ownership, bed size, market competition, and LTCH locations, the association between RN staffing levels and inpatient outcomes was analyzed. The results are summarized in Table 2. As the number of patients per RN increased, the proportions of inpatients with improved moderate-to-severe pain, ability to perform daily activities, pressure ulcers, and return to the community decreased. Conversely, the proportions of inpatients with indwelling urinary catheters and new pressure ulcers increased. The proportion of inpatients with weight loss was not significantly affected by RN staffing levels.

### 3.3. Inpatient Outcomes by Disease Classification Groups According to RN Staffing Levels in LTCHs

After controlling for ownership, bed size, market competition, and LTCH locations, the analysis of inpatients by disease classification groups according to RN staffing levels is presented in Table 3.

In the neurological classification group, as the number of inpatients per RN increased, the proportions of inpatients with improved moderate-to-severe pain, ability to perform daily activities, pressure ulcers, and return to the community decreased. Conversely, the proportions of inpatients with new pressure ulcers and those experiencing weight loss increased. The proportion of inpatients with indwelling urinary catheters was not significantly associated with RN staffing levels.

In the dementia classification group, as the number of inpatients per RN increased, the proportion of inpatients with improved moderate-to-severe pain, improved ability to perform daily activities, improved pressure ulcers, and those returning to the community decreased. Conversely, the proportions of inpatients with indwelling urinary catheters, new pressure ulcers, and experiencing weight loss increased.

As the number of inpatients per RN increased in the infection classification group, the proportions of inpatients with improved moderate-to-severe pain, experiencing weight loss, and those who returned to the community decreased. Conversely, the proportions of inpatients with indwelling urinary catheters and improved pressure ulcers increased. However, the proportion of inpatients with an improved ability to perform daily activities and developing new pressure ulcers were not significantly associated with RN staffing levels.

In the musculoskeletal classification group, as the number of inpatients per RN increased, the proportions of inpatients with improved moderate-to-severe pain, ability to perform daily activities, pressure ulcers, and return to the community decreased. Conversely, the proportions of inpatients with indwelling urinary catheters, new pressure ulcers, and experiencing weight loss increased.

## 4. Discussion

This study assessed RN staffing levels in 1243 LTCHs in the Republic of Korea and examined the association between RN staffing levels and inpatient outcomes by disease classification groups.

The analysis of RN staffing levels in the 1243 LTCHs in the Republic of Korea revealed that in 2021, the average number of inpatients per RN was 9.95. Compared to 2008 (15.4 inpatients per RN) [21] when the differential inpatient fee system was introduced, and 2019 (10.3 inpatients per RN) [22] when the policy to reduce nurse staffing classification standards and provide incentives for a skill mix of two-thirds or more was implemented, the number of patients per RN has gradually decreased. This suggests that policies targeting LTCHs have been effective. However, RN staffing levels are still insufficient, as only approximately 60% of the required legal RN staffing standards in Korean LTCHs (six inpatients per RN) are met.

In this study, the Herfindahl-Hirschman Index ranged from a minimum of 43.91 to a maximum of 2648.79, indicating a significant variation in the level of competition among LTCHs within the regions. This is likely related to the RN staffing levels. Generally, a Herfindahl-Hirschman Index exceeding 2500 indicates very high market concentration, suggesting that a few LTCHs in the area hold a large market share, leading to less competition and a potential monopolistic tendency. A previous study showed that for every 1% increase in the Herfindahl-Hirschman Index, RN staffing intensity decreases by 5% and the RN to non-RN ratio decreases by 4.4% [23]. This finding supports the findings of the present study. LTCHs with high Herfindahl-Hirschman Index might not secure sufficient RN staffing, potentially substituting up to two-thirds of the required nursing staff with Certified Nurse Assistants as permitted by medical law.

In the association between RN staffing levels and inpatient outcomes, without considering the severity of inpatients in LTCHs, higher RN-to-inpatient ratios were associated with a decrease in positive inpatient outcomes such as improvement in pain, ADL, pressure ulcers, and discharge back to the community. Conversely, negative inpatient outcomes such as the presence of indwelling urinary catheters and the incidence of new pressure ulcers increased. The overall findings were consistent with a recent review of the relationship between nurse staffing and quality of care in long-term care settings [24]. However, weight loss was not significantly associated with RN staff levels.

An additional analysis by disease classification group was conducted to consider the inpatient case mix. The disease groups representing >10% of all cases were analyzed. The results showed that in the dementia and musculoskeletal classification groups, higher RN-to-inpatient ratios were associated with lower positive inpatient outcomes, such as discharge back to the community and improvement in pain, ADLs, and pressure ulcers. Higher RN-to-inpatient ratios were also associated with higher negative inpatient outcomes such as improvement in pain and ADL, and higher negative inpatient outcomes such as the presence of indwelling urinary catheters, new pressure ulcers, and weight loss. The neurological group showed similar results, except for the presence of indwelling urinary catheters, which was not significantly associated with RN staffing levels. This result appeared to be related to the calculation indicators for patients with indwelling urinary catheters [25]. The indicator for these patients was an integrated measure that reflected the ratio of high-to low-risk patients. The high-risk group included patients with uncontrolled bowel movements, stage 3 pressure ulcers, a comatose state, a need for total assistance in all ADL, as well as those with quadriplegia, paraplegia, or spinal cord injuries. Damage or lesions to the nervous system can lead to lower urinary tract dysfunction, known as neurogenic bladder, which often requires indwelling urinary catheters [26]. In this study, the neurological disease group mainly included patients with stroke and Parkinson’s disease. Management of voiding dysfunction in patients with neurological diseases such as Parkinson’s disease, cerebrovascular disease, and spinal cord injury may necessitate the consideration of intermittent or indwelling catheterization [27]. The use of catheters is influenced by the nature of the neurological disease, regardless of the level of RN staffing. This underscores the importance of considering disease characteristics when interpreting the impact of RN staffing on inpatient outcomes and highlights the necessity of accounting for the appropriate level of nurse staffing based on nursing needs across disease characteristics.

However, different results were observed in the infection group. As the number of patients per RN increased, the proportion of patients with improved pain and those returning to the community decreased, whereas the proportion of patients with indwelling catheters increased, showing results similar to those of the other groups. However, improvements in ADL and the incidence of new pressure ulcers were not significant. Notably, the improvement in pressure ulcers increased and the proportion of patients experiencing weight loss decreased, demonstrating different outcomes compared to the other disease groups. To understand these differences, a detailed analysis of the diseases within the infection classification group was performed. U07 (emergency use) was the most frequently reported in LTCHs, accounting for 39.2% of the cases. This is likely due to the use of data from 2021 during the coronavirus disease (COVID-19) pandemic. According to health insurance medical care statistics, the largest number of patients were diagnosed with “U07 Emergency Use” [28]. This refers to emergency care for COVID-19 and is a code assigned when COVID-19 is confirmed through laboratory tests, regardless of the clinical signs or severity of symptoms [20]. Therefore, it is believed that the infection group, which contains the highest number of cases with this code, showed different results in patient outcomes that reflect quality indicators for long-term care patients, such as improvements in ADL and pressure ulcers, the incidence of new pressure ulcers, and weight loss. The inconsistency in the association between RN staffing and inpatient outcomes in this group may be attributed to the temporary impact of the COVID-19 pandemic. This infection classification group was not present in the previous study [19]. Therefore, we recommend future research using data from post-pandemic periods to compare trends and validate findings. This will help isolate the pandemic’s effects from other factors influencing RN staffing and inpatient outcomes.

Nevertheless, our study aligns with the findings of previous research that confirmed that the impact of RN staffing levels on patient outcomes varies according to disease severity and nursing needs of diseases [16]. In the United Kingdom, the Shelford Group, composed of 10 major university hospitals of the National Health Service, uses the Safer Nursing Care Tool to classify patients in acute hospitals based on their nursing needs, and periodically provides nurse staffing standards for each classification group [29,30]. This highlights the importance of establishing policies to calculate RN staffing levels for inpatients in LTCHs, taking into account the different nursing needs and severity of the disease classification groups.

This study used nationwide data to analyze the association between RN staffing levels and inpatient outcomes in LTCHs in the Republic of Korea. The key findings indicated that higher RN staffing levels in LTCHs were associated with an increase in positive inpatient outcomes and a decrease in negative inpatient outcomes. These associations were particularly evident for indicators such as pain improvement, pressure ulcer improvement, and community reintegration. These results are consistent with those of previous studies conducted in acute care hospitals, reaffirming that adequate nurse staffing positively impacts patient management and outcomes. The positive association between RN staffing levels and inpatient outcomes has important implications for the development of workforce policy in LTCHs. Specifically, ensuring adequate RN staffing can improve inpatient health outcomes and reduce healthcare costs. This underscores the importance of recognizing the significance of RN staffing for policymakers and hospital administrators, and can aid in developing strategies to optimize RN staffing levels in LTCHs.

This study had several limitations. First, its cross-sectional design made it difficult to establish causal relationships. Future research should adopt a longitudinal design to provide stronger evidence of causation between nurse staffing levels and inpatient outcomes. This approach could involve incorporating advanced statistical methods, such as mixed-effects modeling, could help control for time-varying confounders, improve the robustness of findings, and monitor inpatient outcomes over time in LTCHs with varying staffing levels to determine whether increases in RN staffing lead to measurable improvements in outcomes. Second, the study was constrained by limited access to data, relying solely on publicly available hospital-level datasets accessible to the public. Additionally, we included as many relevant hospital- and market-level factors as possible in our analysis to provide a robust evaluation of the association of RN staffing and inpatient outcomes. However, since this study was conducted at the institutional level across LTCHs nationwide, it did not sufficiently adjust for patient-level characteristics, necessitating caution when generalizing the results. While it would have been ideal to consider patient-level characteristics such as age, sex, and severity which could significantly affect outcomes, these constraints required an alternative approach. To address this, we conducted analyses based on disease classification groups. However, future research could provide a deeper understanding by examining how specific disease groups, such as neurological diseases and musculoskeletal disorders, interact with RN staffing levels to influence outcomes. Third, since this study was limited to LTCHs in the Republic of Korea, further research is needed to determine whether the findings are applicable to other countries or healthcare settings. Additionally, comprehensive analyses that include various variables are necessary, and similar studies should be conducted in other countries or healthcare settings to verify the generalizability of the findings.

This study used market-related factors as control variables, but future research is needed to examine the impact of regional differences or market competition on variations in RN staffing levels, as well as how these factors influence the relationship between RN staffing and inpatient outcomes. There is currently no nationwide data on patient-reported outcomes, such as inpatient satisfaction or quality of life, for LTCH patients in the Republic of Korea. However, developing quality indicators that incorporate these variables in the future and studying the relationship between nurse staffing levels and patient-reported outcomes would be meaningful. In addition, gathering qualitative feedback from patients or their family members could further enhance understanding by providing a more holistic perspective on care quality in LTCHs. In the future, studies could investigate nurse staffing levels in LTCHs in countries with aging trends similar to the Republic of Korea and compare their relationship with inpatient outcomes. Such research could provide critical evidence for addressing the needs of aging populations worldwide and better elucidate the relationship between nurse staffing levels and inpatient outcomes, which can make a practical contribution to policy development.

## 5. Conclusions

This study highlights the critical importance of adequate RN staffing in improving inpatient outcomes in Korean LTCHs. Using comprehensive national data, we identified significant associations between higher patient-to-RN ratios and worsened patient outcomes, including lower improvement rates in moderate to severe pain, ADL, and pressure ulcers, reduced community return rates, increased retention of indwelling urinary catheters, and a higher incidence of new pressure ulcers. These findings have practical implications for healthcare policy and management. Establishing minimum RN staffing standards and providing incentives for hospitals to achieve these levels can enhance the quality of care and patient safety. Future research should focus on exploring the underlying mechanisms linking nurse staffing levels to specific outcomes, as well as evaluating the cost-effectiveness of interventions aimed at optimizing staffing in LTCHs. Addressing these gaps will help policymakers and healthcare administrators achieve sustainable improvements in the care of aging populations in the Republic of Korea, which is transitioning to a super-aged society at one of the fastest rates in the world.

## Figures and Tables

**Table 1:** Characteristics of hospitals, nurse staffing levels, and inpatient outcomes. (*n* = 1243).

Category	Variables	*n* (%) or M ± SD	Min–Max
Hospital-related Factors			
Ownership	Non-private	615 (49.5)	
	Private	628 (50.5)	
Bed size		194.57 ± 100.78	30–2055
	<100	132 (10.6)	
	100-<200	699 (56.2)	
	200-	412 (33.2)	
Inpatients % of daily activities	(Level 1) independent	9.64 ± 20.15	0–100
	(Level 2) need some assistance	43.85 ± 14.47	0–83.7
	(Level 3) entirely dependent	46.47 ± 16.60	0–99.3
Evaluation grade	Grade 1	218 (17.5)	
	Grade 2	408 (32.8)	
	Grade 3	325 (26.2)	
	Grade 4	197 (15.9)	
	Grade 5	95 (7.6)	
Frequently reported diseases	Dementia in AD	1064 (28.0)	
	Hemiplegia	439 (11.6)	
	Cerebral infarction	413 (10.9)	
	Parkinson’s disease	205 (5.4)	
	Emergency use of U07	168 (4.4)	
Market-related Factor			
Herfindahl-Hirschman Index		163.80 ± 192.79	43.91–2648.79
Location	Capital area	417 (33.6)	
	Metropolitan area	346 (27.8)	
	Other regions	480 (38.6)	
Nurse Staffing Level			
Number of inpatients per RN		9.95 ± 2.90	2.60–29.20
Number of inpatients per nurse staffing (RN + CNA)		3.99 ± 0.43	1.00–8.70
Inpatient outcomes (%)			
Improved pain		66.25 ± 26.97	0–100
Improved activities of daily living		28.91 ± 25.23	0–100
Indwelling urinary catheter		3.62 ± 6.58	0–74.4
New pressure ulcer		0.10 ± 0.26	0–2.6
Improved pressure ulcer		39.69 ± 22.43	0–100
Weight loss		0.37 ± 1.13	0–14.9
Community return		48.92 ± 16.85	0–100

AD: Alzheimer’s Disease; CNA: Certified Nurse Assistant; RN: Registered Nurse.

**Table 2:** RN staffing levels and inpatient outcomes. (*n* = 1243).

Variables	Crude Model					Adjusted Model				
	B	SE	95% CI		*p*	B	SE	95% CI		*p*
Improved pain (*n* = 927)	−0.015	0.002	−0.018	−0.012	<0.001	−0.015	0.002	−0.018	−0.012	<0.001
Improved ADL (*n* = 1227)	−0.032	0.002	−0.036	−0.028	<0.001	−0.015	0.002	−0.019	−0.011	<0.001
Indwelling UC (*n* = 1242)	0.020	0.005	0.010	0.031	<0.001	0.038	0.005	0.029	0.048	<0.001
New PU (*n* = 1197)	0.090	0.030	0.031	0.149	0.003	0.082	0.027	0.029	0.134	0.002
Improved PU (*n* = 1099)	−0.007	0.002	−0.011	−0.004	<0.001	−0.007	0.002	−0.011	0.004	<0.001
Weight loss (*n* = 1234)	−0.014	0.017	−0.047	0.020	0.417	0.007	0.015	−0.023	0.037	0.642
Community return (*n* = 981)	−0.025	0.002	−0.029	−0.022	<0.001	−0.019	0.002	−0.023	−0.016	<0.001

ADL: Activities of Daliy Living; PU: Pressure Ulcer; UC: Urinary Catheter.

**Table 3:** RN staffing levels and inpatient outcomes by classification of disease groups.

	Variables	Crude Model					Adjusted Model				
		B	SE	95% CI		*p*	B	SE	95% CI		*p*
Neurological (*n* = 849)	Improved pain (*n* = 639)	−0.014	0.002	−0.018	−0.011	<0.001	−0.024	0.002	−0.027	−0.020	<0.001
	Improved ADL (*n* = 849)	−0.035	0.002	−0.040	−0.031	<0.001	−0.026	0.003	−0.031	−0.022	<0.001
	Indwelling UC (*n* = 849)	−0.016	0.007	−0.029	−0.003	0.019	−0.002	0.007	−0.014	0.011	0.820
	New PU (*n* = 846)	0.101	0.037	0.028	0.173	0.006	0.087	0.033	0.022	0.153	0.009
	Improved PU (*n* = 793)	−0.005	0.002	−0.009	−0.001	0.017	−0.010	0.002	−0.014	−0.005	<0.001
	Weight loss (*n* = 846)	0.094	0.020	0.055	0.134	<0.001	0.072	0.018	0.037	0.107	<0.001
	Community return (*n* = 666)	−0.035	0.002	−0.039	−0.031	<0.001	−0.038	0.002	−0.042	−0.034	<0.001
Dementia	Improved pain (*n* = 796)	−0.015	0.002	−0.019	−0.012	<0.001	−0.018	0.002	−0.022	−0.015	<0.001
(*n* = 1075)	Improved ADL (*n* = 1074)	−0.031	0.002	−0.036	−0.027	<0.001	−0.015	0.002	−0.020	−0.011	<0.001
	Indwelling UC (*n* = 1075)	0.013	0.006	0.001	0.024	0.034	0.025	0.006	0.014	0.159	<0.001
	New PU (*n* = 1072)	0.109	0.032	0.045	0.172	<0.001	0.102	0.029	0.045	0.13	0.002
	Improved PU (*n* = 990)	−0.010	0.002	−0.013	−0.006	<0.001	−0.007	0.002	−0.011	−0.003	0.001
	Weight loss (*n* = 1071)	0.071	0.019	0.033	0.108	<0.001	0.067	0.017	0.034	0.100	<0.001
	Community return (*n* = 822)	−0.030	0.002	−0.034	−0.027	<0.001	−0.029	0.002	−0.033	−0.025	<0.001
Infection	Improved pain (*n* = 299)	−0.005	0.003	−0.010	0.001	0.087	−0.006	0.003	−0.012	0.000	0.035
(*n* = 388)	Improved ADL (*n* = 388)	−0.026	0.003	−0.032	−0.019	<0.001	0.002	0.004	−0.005	0.009	0.574
	Indwelling UC (*n* = 388)	−0.007	0.009	−0.025	0.011	0.434	0.031	0.009	0.013	0.049	0.001
	New PU (*n* = 387)	0.065	0.053	−0.038	0.169	0.217	0.079	0.051	−0.021	0.178	0.120
	Improved PU (*n* = 365)	−0.007	0.003	−0.013	−0.001	0.033	0.007	0.003	0.000	0.013	0.044
	Weight loss (*n* = 385)	−0.143	0.037	−0.215	−0.071	<0.001	−0.115	0.038	−0.190	−0.039	0.003
	Community return (*n* = 303)	−0.039	0.003	−0.045	−0.034	<0.001	−0.036	0.003	−0.043	−0.029	<0.001
Musculoskeletal	Improved pain (*n* = 276)	−0.029	0.003	−0.035	−0.024	<0.001	−0.025	0.003	−0.031	−0.019	<0.001
(*n* = 363)	Improved ADL (*n* = 362)	−0.042	0.004	−0.050	−0.035	<0.001	−0.013	0.004	−0.021	−0.006	<0.001
	Indwelling UC (*n* = 363)	0.054	0.009	0.035	0.072	<0.001	0.058	0.008	0.042	0.074	<0.001
	New PU (*n* = 363)	0.140	0.053	0.035	0.244	0.009	0.126	0.043	0.042	0.210	0.003
	Improved PU (*n* = 331)	−0.025	0.004	−0.031	−0.018	<0.001	−0.015	0.004	−0.022	0.008	<0.001
	Weight loss (*n* = 361)	0.162	0.030	0.103	0.220	<0.001	0.144	0.029	0.087	0.200	<0.001
	Community return (*n* = 278)	−0.026	0.003	−0.032	−0.020	<0.001	−0.019	0.003	−0.025	−0.012	<0.001

ADL: Activities of Daliy Living; PU: Pressure Ulcer; UC: Urinary Catheter.

## Data Availability

The datasets used and/or analyzed during the current study available from the corresponding author on reasonable request.

## Supplementary Materials

The following supporting information can be downloaded at: https://www.mdpi.com/article/10.3390/healthcare12242509/s1, Table S1: The variables used for analysis and their definitions; Table S2: Classification groups of frequently reported diseases in LTCHs.
