# Supplementary material for: Registered Nurse Staffing and Inpatient Outcomes in Korean Long-Term Care Hospitals
Source: Healthcare (Basel). 2024 Dec 11;12(24):2509. doi: 10.3390/healthcare12242509 (PMC11728238; doi:10.3390/healthcare12242509)
Supplement: Supplementary file 1 [file healthcare-12-02509-s001.zip › healthcare-3269759-supplementary.pdf]

Table S1. The variables used for analysis and their definitions.

Category	Variables	Operational Definition
Independent variable	Number of inpatients per RN	Average number of inpatients per registered nurse during the study period
Dependent variables	Improved pain	Percentage of inpatients who showed improvement in moderate to severe pain compared to the previous month
	Improved activities of daily living	Percentage of inpatients who showed improvement in activities of daily living (ADL) compared to the previous month
	Indwelling urinary catheter	Percentage of inpatients with indwelling urinary catheters
	New pressure ulcer	Percentage of inpatients who developed newly pressure ulcers compared to the previous month
	Improved pressure ulcer	Percentage of inpatients who showed improvement in pressure ulcers compared to the previous month
	Weight loss	Percentage of inpatients who showed more than a 5% weight loss compared to the previous month
	Community return	Percentage of inpatients who were discharged to their home or a facility
Control variables	Ownership	Non-private: public, corporation, consumer cooperative<br>Private
	Bed size	The total number of general and premium beds
	Herfindahl-Hirschman Index	Squaring the proportion of the hospital's beds relative to the total number of beds in the market and then summing these squared values.
	Location	Capital Region: Seoul, Incheon, Gyeonggi<br>Metropolitan Cities: Busan, Daegu, Gwangju, Daejeon, Ulsan<br>Other regions: Gangwon, Chungbuk, Chungnam, Jeonbuk, Jeonnam, Gyeongbuk, Gyeongnam, Jeju, Sejong

Table S2. Classification groups of frequently reported diseases in LTCHs (n = 3785).

Classification	Disease	n (%)
Neurological	Cerebral infarction,<br>Cerebral palsy,<br>Hemiplegia,<br>Intracerebral haemorrhage,<br>Other disorders of nervous system, NEC,<br>Paraplegia and tetraplegia,<br>Parkinson's disease,<br>Sequelae of cerebrovascular disease,<br>Spinal muscular atrophy and related syndromes,<br>Status epilepticus	1164 (30.8)
Dementia	Alzheimer's disease,<br>Dementia in Alzheimer's disease,<br>Unspecified dementia,<br>Vascular dementia	1146 (30.3)
Infection	Bacterial pneumonia, NEC,<br>Emergency use of U07,<br>Intracranial and intraspinal abscess and granuloma,<br>Other sepsis,<br>Pneumonia, organism unspecified,<br>Pneumonitis due to solids and liquids,<br>Resistance to betalactam antibiotics,<br>Resistance to other antibiotics,<br>Special screening examination for infectious and parasitic diseases,<br>Viral infection of unspecified site	429 (11.3)
Musculoskeletal	Abnormal involuntary movements,<br>Calcification and ossification of muscle,<br>Dislocation, sprain and strain of joints and ligaments at ankle and foot level,<br>Dislocation, sprain and strain of joints and ligaments of lumbar spine and pelvis,<br>Fracture of femur,<br>Fracture of lumbar spine and pelvis,<br>Gonarthrosis [arthrosis of knee],<br>Other arthritis,<br>Other arthrosis,<br>Other disorders of muscle,<br>Other joint disorders, NEC,<br>Other soft tissue disorders, NEC,<br>Other specific joint derangements,<br>Other spondylopathies,<br>Polyarthrosis,<br>Shoulder lesions,<br>Soft tissue disorders related to use, overuse and pressure,<br>Spondylosis	391 (10.3)
Cancer	Malignant neoplasm of breast,<br>Malignant neoplasm of bronchus and lung,<br>Malignant neoplasm of cervix uteri,<br>Malignant neoplasm of colon,<br>Malignant neoplasm of corpus uteri,<br>Malignant neoplasm of ovary,	255 (6.7)

	Malignant neoplasm of pancreas,<br>Malignant neoplasm of rectum,<br>Malignant neoplasm of stomach,<br>Malignant neoplasm of thyroid gland,	
Urinary	Chronic kidney disease,<br>Other disorders of urinary system,<br>Other symptoms and signs involving the urinary system,<br>Unspecified urinary incontinence-	132 (3.5)
Pain	Dorsalgia,<br>Headache	114 (3.0)
Korean or Oriental Medicine	Disease Name of Korean Medicine or Disease Pattern/syndrome of Oriental Medicine:<br>Heart and kidney yang deficiency pattern,<br>Heart deficiency patterns,<br>Heart excess patterns,<br>Kidney patterns,<br>Manic disorder,<br>Neck stiffness disorder,<br>Numbness disorder,<br>Post COVID-19 condition,<br>Principle-base patterns,<br>Qi patterns<br>Sequela of wind stroke disorder<br>Spleen and stomach yang deficiency pattern<br>Spleen patterns	47 (1.3)
Cerebrovascular	Acute myocardial infarction,<br>Essential(primary) hypertension,<br>Other and unspecified disorders of circulatory system	43 (1.1)
Gastrointestinal	Dysphagia,<br>Functional dyspepsia,<br>Gastritis and duodenitis,<br>Hepatic failure, NEC,<br>Nausea and vomiting,	13 (0.4)
Endocrine: diabetes	Type 2 diabetes mellitus,<br>Unspecified diabetes mellitus	12 (0.3)
General symptoms	Symptoms, signs and abnormal clinical and laboratory findings, NEC: General symptoms and signs<br>- Dizziness and giddiness,<br>- Lack of expected normal physiological development,<br>- Malaise and fatigue,<br>- Senility,<br>- Somnolence, stupor and coma	11 (0.3)
Skin	Decubitus ulcer and pressure area,<br>Disturbances of skin sensation	9 (0.2)
Etc1	Persons encountering health services for examination and investigation:<br>General examination and investigation of persons without complaint and reported diagnosis<br>Medical observation and evaluation for suspected diseases and conditions, ruled out<br>Other specified prophylactic measures	9 (0.2)

Etc2	Intracranial injury, Mild mental retardation, Other mental disorders due to brain damage and dysfunction and to physical disease, Unspecified mental retardation	7 (0.2)
Respiratory 12	Abnormalities of breathing,<br>Other chronic obstructive pulmonary disease	3 (0.1)

The list of disease in S2 is alphabetical order. Some hospitals did not provide this information.
